# Persistence of bacterial and archaeal communities in sea ice through an Arctic winter

**DOI:** 10.1111/j.1462-2920.2010.02179.x

**Published:** 2010-07

**Authors:** R Eric Collins, Gabrielle Rocap, Jody W Deming

**Affiliations:** School of OceanographyBox 357940, 1503 NE Boat St., University of WashingtonSeattle, WA 98195, USA

## Abstract

The structure of bacterial communities in first-year spring and summer sea ice differs from that in source seawaters, suggesting selection during ice formation in autumn or taxon-specific mortality in the ice during winter. We tested these hypotheses by weekly sampling (January–March 2004) of first-year winter sea ice (Franklin Bay, Western Arctic) that experienced temperatures from −9°C to −26°C, generating community fingerprints and clone libraries for *Bacteria* and *Archaea*. Despite severe conditions and significant decreases in microbial abundance, no significant changes in richness or community structure were detected in the ice. Communities of *Bacteria* and *Archaea* in the ice, as in under-ice seawater, were dominated by SAR11 clade *Alphaproteobacteria* and Marine Group I *Crenarchaeota*, neither of which is known from later season sea ice. The bacterial ice library contained clones of *Gammaproteobacteria* from oligotrophic seawater clades (e.g. OM60, OM182) but no clones from gammaproteobacterial genera commonly detected in later season sea ice by similar methods (e.g. *Colwellia*, *Psychrobacter*). The only common sea ice bacterial genus detected in winter ice was *Polaribacter*. Overall, selection during ice formation and mortality during winter appear to play minor roles in the process of microbial succession that leads to distinctive spring and summer sea ice communities.

## Introduction

Arctic sea ice covers an area of about 15 × 10^6^ km^2^ at its maximal extent during winter (Antarctic 18 × 10^6^ km^2^; Fetterer and Knowles, 2002), providing an extensive (albeit shrinking; Serreze *et al.*, 2007) habitat for microorganisms. During the annual lifetime of polar sea ice, it experiences wide ranges in environmental conditions, yet changes in its microbial (bacterial and archaeal) communities through the seasons are not well known (Mock and Thomas, 2005; Deming, 2009). Winter sea ice in the Arctic is an extreme environment characterized by limited light, very cold temperatures in its upper horizons (to −35°C) and correspondingly high salinity (37–237‰) in its brine inclusions, where the organisms have been observed to reside (Junge *et al.*, 2001). The assumption that these microbial inhabitants are largely inactive during the winter has been tested infrequently. Working with first-year sea ice north of Barrow (Alaska) during the coldest month of the year (March), Junge and colleagues (2004) observed that a small percentage of cells (0.5–4.0% by CTC staining) were metabolically active to −20°C, the coldest ice horizon examined. Wells and Deming (2006b), working with landfast sea ice in Franklin Bay (Western Arctic, also in March), detected an increase in bacterial numbers (doubling time > 4 days) in one of the three ice-brine samples they incubated at −12°C (salinity of 160‰).

Relative to other marine environments, bacteria in sea ice have proven highly amenable to cultivation: up to 50% of total counts from Antarctic ice (Helmke and Weyland, 1995) and 62% from Arctic ice (Junge *et al.*, 2002). The bacterial groups that have been cultured from polar sea ice consist primarily of *Gammaproteobacteria* of the orders *Oceanospirillales* and *Alteromonadales*, the marine *Roseobacter* clade of *Alphaproteobacteria* (Bowman *et al.*, 1997; Brown and Bowman, 2001; Brinkmeyer *et al.*, 2003; Buchan *et al.*, 2005), and the *Bacteroidetes* (also known as *Cytophaga*–*Flavobacter*–*Bacteroides*). *Betaproteobacteria* and high G+C Gram-positives (*Actinobacteria*) have also been obtained in culture from Antarctic sea ice (Junge *et al.*, 1998) and Baltic Sea ice (Kaartokallio *et al.*, 2005). Results from culture-independent methods, including cloning and sequencing of 16S rRNA genes and fluorescent *in situ* hybridization (FISH), overlap remarkably well with culture-based results, confirming the prevalence of these bacterial groups in spring and summer sea ice (Brown and Bowman, 2001; Brinkmeyer *et al.*, 2003). In contrast, *Archaea* were not known from sea ice until recently, even though they are prevalent members of Arctic pelagic communities (Galand *et al.*, 2006; 2008b; Wells *et al.*, 2006) and comprise a sizable fraction of the biomass in Antarcticfrazil ice in late winter (Delong *et al.*, 1994). To date, *Archaea* have only been detected in Arctic winter sea ice using domain-level probes, where they comprised ≤ 3.4% of the total assemblage (Junge *et al.*, 2004). They appear absent in sea ice of later seasons by both culture-based and culture-independent methods (Brown and Bowman, 2001; Brinkmeyer *et al.*, 2003).

Possible shifts in community composition and richness in Arctic winter ice over time and increasingly severe conditions are not known. From cultivation studies of Antarctic sea ice, psychrophilic bacteria are thought to outlive psychrotolerant species during winter (Helmke and Weyland, 1995; Delille and Rosiers, 1996; Helmke and Weyland, 2004; Fiala *et al.*, 2006), leaving them positioned to dominate during the biologically productive spring and summer months. Such common heterotrophic microorganisms may also begin their habitation of sea ice in relative abundance if attached to larger phytoplankton known to entrain selectively into ice by frazil ice scavenging (Grossmann, 1994). The dynamics of microbial communities in polar sea ice, whether during or after ice entrapment, have not been examined using phylogenetic approaches, although these techniques have been used to study winter succession of bacterial and archaeal communities in polar waters (Murray *et al.*, 1998; Murray and Grzymski, 2007), including surface waters at our study site (Alonso-Sáez *et al.*, 2008). The only ice examined by a PCR-based ‘fingerprinting’ technique for high-throughput analysis of microbial community structure (Osborn *et al.*, 2000; Hewson *et al.*, 2007; Fuhrman *et al.*, 2008) is the warm (> −5°C) and thin (< 30 cm) ice of the brackish (5–6‰) Baltic Sea (Kaartokallio *et al.*, 2008). There the dynamics of *Alphaproteobacteria*, *Gammaproteobacteria*, and *Bacteroidetes* over the short (2 month) lifetime of this ‘mild’ ice were linked to exchange processes at the ice–water interface and progression of the ice-algal bloom. These features do not pertain to upper horizons of much colder, thicker and light-limited Arctic winter sea ice, the subject of this study.

During the Canadian Arctic Shelf Exchange Study (CASES), when the CCGS *Amundsen* was immobilized in landfast sea ice of Franklin Bay (Western Arctic) through the winter, we first investigated the spatial heterogeneity and temporal dynamics of particulate matter, including microorganisms and particulate extracellular polymeric substances (pEPS), within the ice (Collins *et al.*, 2008). The same ice field was cored repeatedly each week from January through March, a period when ice thickness increased from 90 to 200 cm and upper ice temperatures were well below −5°C, leaving the ice impermeable (Golden *et al.*, 1998) and the microbial communities entrapped. We focused on the upper 70 cm of the ice, analysing three 10 cm horizons centred at 25, 45 and 65 cm below the ice surface (reserving subsamples for later phylogenetic analyses). The results indicated winter losses of 38% and 49% of the total number of bacteria in the two coldest ice horizons (Fig. S1), where *in situ* temperatures had ranged from −15°C to −26°C (25 cm) and −12°C to −22°C (45 cm; the range at 65 cm was −9°C to −18°C; Collins *et al.*, 2008). At these temperatures brine salinities are also extreme, from 130‰ to 230‰. We attributed some of the bacterial losses to virally mediated mortality, given measurements of viral production in ice brines from the same ice field (Wells and Deming, 2006b). We attributed the persistence of the majority of bacteria throughout the ice to the generic cryoprotective effects of pEPS (Krembs and Deming, 2008), which had increased significantly in all three horizons during winter (Collins *et al.*, 2008).

Here, we investigate effects of the increasingly severe conditions in the aforementioned sea ice on the structure and richness of the natural microbial communities found within it, beginning several weeks after ice formation. Because bacterial communities that thrive within spring and summer sea ice differ substantially from pelagic communities prior to freeze-up, we hypothesized that extreme conditions in winter sea ice would exert selective pressure on the microbial community, favouring survival and subsequent dominance of the easily cultured psychrophilic *Bacteria* already known from spring and summer sea ice. To test this hypothesis we performed community fingerprinting of *Bacteria* (by automated ribosomal intergenic spacer analysis, ARISA) and *Archaea* (by terminal restriction fragment length polymorphism, T-RFLP) on the entire sample set collected during the CASES overwintering expedition (Collins *et al.*, 2008), also generating complementary clone libraries of bacterial and archaeal 16S rRNA genes from selected samples of winter sea ice and under-ice seawater. Although we were unable to test directly for selection during the autumn freezing process, our results also bear upon this concept.

## Results

### Community dynamics

#### Bacterial ARISA

**Fig. 1:**
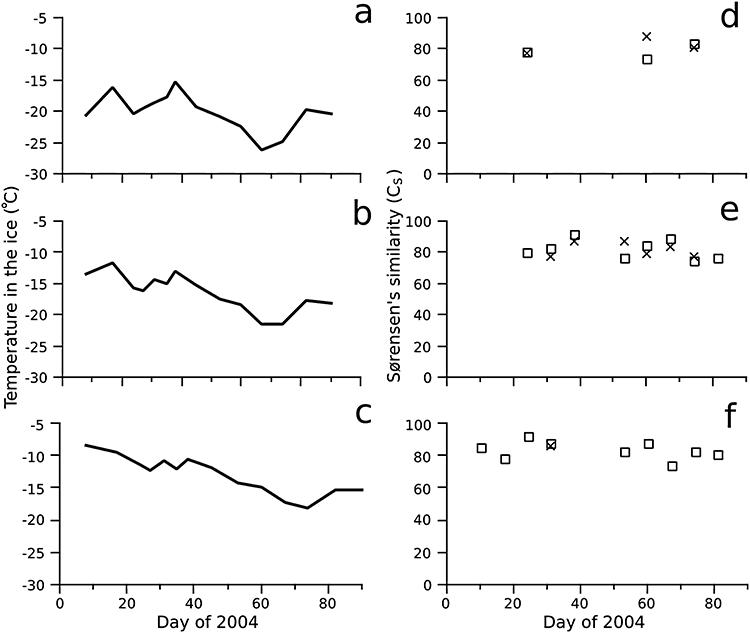
Temperature in the ice (A–C) from Collins and colleagues (2008), and pairwise similarities (D–F) for bacterial (×) and archaeal (□) communities in Arctic winter sea ice horizons I–III, representing depths of 25, 45 and 65 cm below the ice surface (top, middle and bottom panels respectively). Sørensen's similarity index (%) was calculated for each ARISA (*Bacteria*) or T-RFLP (*Archaea*) sample relative to the first sample in the time series, 24-II (*Bacteria*) or 17-II (*Archaea*), which each had self-similarities of 100% (unplotted). Pearson correlation coefficients of pairwise similarity over time were not significant (at *P* < 0.05) for either community in any horizon.

Contrary to expectation, no changes in the community structure (Fig. 1) or richness (Fig. S1) of the sea ice bacterial community were detected through the winter. A large majority (77.5%) of the total ARISA signal intensity (i.e. global cumulative peak height) derived from 14 operational taxonomic units (OTUs) present in all 11 successfully analysed sea ice samples (Table 1). Clone libraries of the 16S-intergenic transcribed spacer (ITS)-23S region identified some of these common OTUs, including SAR11 clade *Alphaproteobacteria*, which made up 50% of the total signal intensity (Table 1), in agreement with the dominance of this clade in those clone libraries; OTUs best matching *Polaribacter*, a genus of *Bacteroidetes*, made up a further 12%. Several more OTUs persisting through the winter had best matches to various *Alphaproteobacteria*, *Gammaproteobacteria* and *Flavobacteria* (Table 1). Correlation analysis revealed no significant change (*P* < 0.05) in community structure similarity (Sørensen's index; 84 ± 7%, Fig. 1) or richness (range 19–29, Fig. S1) over time in any horizon. Clustering analysis also indicated no differences among communities by depth horizon, but the limited number and non-uniform distribution of successfully amplified samples precluded further statistical assessments as a function of ice depth. The under-ice seawater sample (88-SW), though lacking about 30% of the OTUs found in every ice sample, was nevertheless dominated by many of the same OTUs detected in the sea ice library (Table 1), yielding Sørensen's similarity index of 64.3% relative to ice sample 24-II.

**Table 1:** Bacterial ARISA OTUs in Franklin Bay (FB) sea ice horizons I–III (representing depths of 25, 45 and 65 cm below the ice surface) and under-ice seawater (SW).

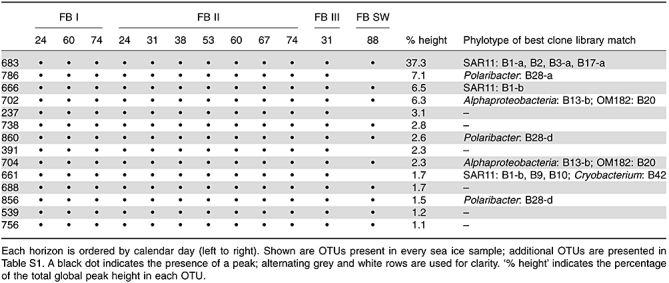

Several OTUs persisting in ice through the winter had no representatives in the bacterial clone library; these OTUs summed to 15% of the total signal, indicating that potentially important groups were missed by this approach. Nevertheless, overlap between clone library sequences and ARISA OTUs (Table 1, Table S1) was substantial. Of the 50 identifiable bacterial subtypes (members of the same phylotype, as identified by 16S rRNA gene sequence, with variable ARISA lengths) detected in the clone libraries, 32 subtypes had predicted ARISA lengths ± 1 bp of an ARISA OTU (Table 1, Table S1); 10 more subtypes had putative matches ± 2.5 bp.

#### Archaeal T-RFLP

Archaeal 16S rRNA genes were readily amplified from these winter sea ice samples, yielding a larger data set than for *Bacteria*, yet still no changes in community structure (Fig. 1) or richness (Fig. S1) of the archaeal communities were detected through the winter. A large majority (88%) of the total T-RFLP signal intensity derived from 17 OTUs present in all 21 successfully analysed sea ice samples (Table 2). Those OTUs included several matching the Marine Group I.1a *Crenarchaeota*, which comprised 45% of the total signal intensity (Table 2), in agreement with the dominance of this clade in the clone libraries (Table 3). OTUs best matching the Marine Group II.b *Euryarchaeota* made up a further 25% of the total signal intensity. Correlation analysis revealed no significant change (*P* < 0.05) over time in community structure similarity (81 ± 6%, Fig. 1) or richness (range 18–33, Fig. S1) in any horizon.

**Table 3:** A summary of the bacterial and archaeal clone libraries from sea ice horizon I (25 cm depth) and under-ice seawater, including the abundance of major taxonomic groups.

	Sea ice	Seawater
Day of year collected	74 + 81	35
Temperature at collection	−22°C	−1.7°C
Bacterial clone libraries	FB04bi	FB04bw
All *Bacteria*	109	46
*Proteobacteria*		
*Alphaproteobacteria*		
SAR11 clade	76	22
Other	5	3
*Gammaproteobacteria*	12	2
*Betaproteobacteria*	2	1
*Deltaproteobacteria*	1	0
Unclassified *Proteobacteria*	1	0
*Bacteroidetes*		
*Flavobacteria*	6	13
*Sphingobacteria*	2	3
Other		
*Actinobacteria*	3	1
*Verrucomicrobia*	1	0
‘Marine Group A’	0	1
Archaeal clone libraries	FB04ai	FB04aw
All *Archaea*	52	45
*Crenarchaeota*		
Marine Group I	46	41
*Euryarchaeota*		
Marine Group II	6	4

**Table 2:** Archaeal T-RFLP OTUs in Franklin Bay (FB) sea ice horizons I–III (representing depths of 25, 45 and 65 cm below the ice surface), and under-ice seawater (SW).

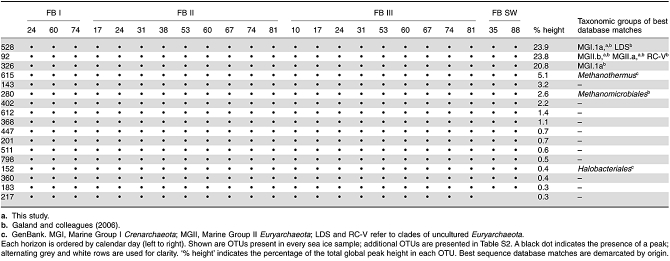

No trend in spatial distribution was observed in the dominant members of the community, but several minor unidentified OTUs exhibited such patterns: best matches to Marine Group I.3a *Crenarchaeota* (515 bp) and *Thermoplasma* (228 bp) were found primarily in upper ice horizons; OTUs with best matches to Marine Group I.1c *Crenarchaeota* (499 bp), *Methanobacteria* (477 bp), RC-V *Euryarchaeota* (531 bp) and unknown groups (529 bp, 322 bp) were found primarily in lower ice horizons (Table 2, Table S2). The under-ice seawater samples, 35-SW and 88-SW, contained most of the same OTUs dominant in the sea ice library (Table 2, Table S2) and did not cluster separately from the ice samples, although some minor OTUs were present only in the seawater samples: 317 bp, 428 bp and 602 bp. Relative to ice sample 17-II, seawater samples 35-SW and 88-SW had Sørensen's similarity indices of 70.8% and 67.8% respectively.

All of the phylotypes detected in the archaeal clone libraries were also detected as T-RFLP OTUs, although many OTUs persisting through the winter had no representatives in our clone libraries, possibly as a result of the different primers used for cloning and fingerprinting. Several of these OTUs were putatively identified using a database of sequences from a recent study (Galand *et al.*, 2006) conducted near the outflow of the Mackenzie River (due west of our study site; Fig. S2), including *Methanomicrobiales*, uncultured methanogen-associated groups LDS and RC-V, and uncultured members of the Marine Group I.3a and Marine Group I.3c *Crenarchaeota*. Other OTUs were putatively identified as halophiles (*Halobacteriales*, 152 bp) or thermophiles (e.g. *Methanothermus*, 615 bp and *Thermoplasmata*, 228 bp), based on a database of predicted terminal restriction fragment lengths of archaeal sequences from GenBank.

### Community composition

#### Bacterial community

The dominant phylotypes in both sea ice and seawater libraries (70% and 48% of sequences respectively; Table 3) were associated with the common seawater clade of SAR11 *Alphaproteobacteria* (Fig. S3), consistent with their dominance in the ARISA analysis (Table 1). Two major SAR11 subtypes were detected; neither showed a differential distribution between the sea ice and seawater libraries (Table S3).

Overlap between the sea ice and seawater libraries was limited: 6 of 44 phylotypes were shared between ice (28 total phylotypes) and water (22 total phylotypes), but due to the common dominance of SAR11, no statistical difference was detected between the libraries using WebLIBSHUFF (*p* = 0.143; *p* = 0.183). Differences were evident in the relative occurrence of *Gammaproteobacteria*, which appeared primarily in the sea ice library, and of *Bacteroidetes*, found primarily in the seawater library (Table S3). The most abundant gammaproteobacterial phylotypes clustered with cultured oligotrophic bacterioplankton clades OM182 and OM60 (Fig. S4, Table S3). The *Bacteroidetes* sequences were dominated by polar marine *Cryomorphaceae* phylotypes, present only in the seawater library. Despite being represented by only five sequences in the libraries, the single *Polaribacter* phylotype consisted of four subtypes. Together, two of these subtypes accounted for 11.2% of the total signal intensity in the ARISA analysis (Table 1). Each library included a different OM43-clade *Methylophilales* phylotype, as well as several distinct marine *Roseobacter* phylotypes (Fig. S3, Table S3). Other unshared phylotypes belonged to the high G+C Gram-positive *Actinobacteria* (sea ice and seawater), *Verrucomicrobia* and *Deltaproteobacteria* (sea ice), and the uncultured ‘Marine Group A’ division (seawater). The seawater library had a greater Chao1 index of richness (174 for seawater, 39 for ice), likely underestimated due to lesser coverage (61% for seawater, 88% for ice), but the libraries had similar Shannon diversity indices (2.25 for seawater, 2.43 for ice).

Eukaryotic 18S rRNA gene sequences were detected only in the seawater library. These 13 sequences were closely related to uncultured marine stramenopile group MAST-1, clade NS1A, with worldwide distribution including Arctic and Antarctic surface waters (Lovejoy *et al.*, 2006; Massana *et al.*, 2006). The predicted ARISA fragment lengths for these sequences were less than 100 bp so their detection by ARISA was not likely.

#### Archaeal community

**Fig. 2:**
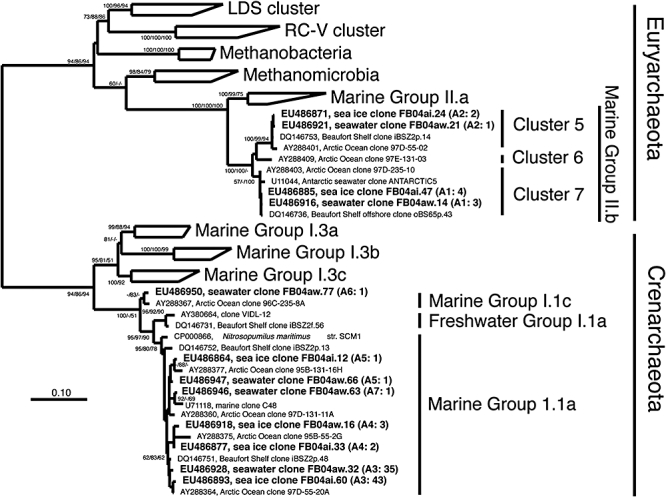
Phylogenetic tree of archaeal 16S rRNA gene sequences. Tree topology was defined by the consensus of 1000 maximum parsimony bootstrap replications utilizing 511 parsimony-informative nucleotides. Branch lengths were defined by Tamura-Nei distances calculated from 807 hypervariable-masked nucleotides. Node values indicate percentage of 10 000 distance, 1000 maximum parsimony and 100 maximum likelihood bootstrap replications respectively; only bootstrap values greater than 50% are shown. One sequence from each phylotype (defined by > 99% similarity) from each library in this study is shown in bold, followed in parentheses by the phylotype and number of sequences from each library within that phylotype.

Archaeal sequences were present in both sea ice and seawater clone libraries; seven phylotypes had high similarities to existing Arctic archaeal clone sequences. The great majority (91%) of sequences from the archaeal libraries belonged to *Crenarchaeota* of the Marine Group I clade, most of which fell into a single phylotype of the Marine Group I.1a (Fig. 2, Table S4). The remaining crenarchaeal sequences were scattered among four more phylotypes within the Marine Group I.1a and Marine Group I.1c clades (Fig. 2). Two phylotypes belonged to uncultured *Euryarchaeota* of the Marine Group II.b clade clusters 5 and 7. The libraries had high coverage (> 95% each), similar richness (5 for ice, 6 for seawater) and similar indices of Shannon diversity (0.61 for ice, 0.76 for seawater).

## Discussion

The expectation that selective losses of dominant bacterial and archaeal community members would occur under the extreme conditions of winter sea ice was not realized in this study. Although both bacterial growth and mortality had been inferred from changes in total bacterial counts (Wells and Deming, 2006b; Collins *et al.*, 2008), microbial community succession was not detectable in the upper ice horizons we sampled. We found no significant change in bacterial or archaeal richness or community similarity over the 3-month period of our investigation (Fig. 1, Fig. S1). Persistence of the dominant members of the sea ice microbial community through winter was also evidenced by the high proportion of total signal associated with members present in every sea ice sample analysed by ARISA (Table 1) and T-RFLP (Table 2). Whereas culture-based studies of heterotrophic microbes had suggested forces at work in sea ice to select for psychrophilic over psychrotolerant strains (Kaneko *et al.*, 1977; Delille and Rosiers, 1996; Delille *et al.*, 1997; Fiala *et al.*, 2006), not even the extreme conditions of the winter sea ice we studied exerted any more than very limited (statistically undetectable) selective pressure at the taxonomic level on the bacterial and archaeal communities entrapped in this ice. Techniques used with polar seawater to quantify differences in relative abundance of specific groups of microorganisms, like FISH (Alonso-Sáez *et al.*, 2008), quantitative PCR of 16S rRNA and functional genes like *amoA* (Galand *et al.*, 2009b), or massively parallel hypervariable tag sequencing (Huse *et al.*, 2008; Galand *et al.*, 2009a), have not yet been applied to sea ice. Their use on winter sea ice might reveal subtle patterns of succession in bacterial and archaeal communities that escaped the sensitivity limits of our methods.

Many *Bacteria* and *Archaea* may lie dormant in winter sea ice (Kaneko *et al.*, 1977; Helmke and Weyland, 1995), surviving without reproducing and thus limiting the possible mechanisms of selection. Although *Bacteria* and *Archaea* were presumed to ‘survive’ if their DNA was present on our membrane filters, we have no data to demonstrate their continued viability *in situ*. Protistan bacterivores, which can selectively graze bacteria based on size and biochemical cues, are essentially absent from very cold Arctic winter ice (Krembs *et al.*, 2002) and so are unlikely to contribute a selective effect, even if they may play a role in shaping the microbial community in warmer ice. The persistence of the ice microbial communities that we measured through winter is consistent with cells being relatively inactive in very cold sea ice and surviving in the absence of bacterivores, whereas more active under-ice seawater communities are subject to predation and do change over the course of winter (Murray and Grzymski, 2007; Alonso-Sáez *et al.*, 2008). Complete preservation in the ice, however, is inconsistent with reports of actively respiring cells in Arctic winter sea ice (Junge *et al.*, 2004) and, from the same ice we sampled, examples of bacterial mortality (Collins *et al.*, 2008) and viral and bacterial production in experimental ice brines (Wells and Deming, 2006b). To reconcile the general absence of species-specific mortality reported here with the presumed presence of a mixture of active and inactive populations (whether taxonomically similar or not), both populations must share similar mortality rates. This assumption can be fulfilled if the primary mechanisms of mortality in the ice are taxonomically non-selective, despite the possibility that they may each affect active and inactive cells differently.

Two mechanisms to consider for non-selective mortality are virally mediated lysis and cell damage from co-occurring extremes in temperature and salinity. Modelled contact rates between viruses and bacteria in sea ice brines are extremely high (up to 600 times that in seawater; Wells and Deming, 2006b) and, under environmental stress, host specificity may give way to a broad range of infectable hosts (Wells and Deming, 2006a), linking both mechanisms under consideration. Viral production (whether by species-specific or generalist phages) may play a role in mortality of any taxonomic group that remains active in the ice, whereas dormant populations might be affected not by viral reproduction from within but ‘lysis from without’ due to a large number of attached viruses (Delbrück, 1940).

For a generalized protective mechanism for cells within sea ice, hydrated coatings of extracellular polymeric substances (EPS) that simultaneously buffer against external ice-crystal damage, osmotic shock and viral attack has been proposed (Krembs *et al.*, 2002; Krembs and Deming, 2008). EPS are produced by many sea ice bacteria in culture (Mancuso Nichols *et al.*, 2005; Marx *et al.*, 2009), colonized by bacteria in sea ice (Meiners *et al.*, 2004), and increased in abundance during winter in the ice we studied (Collins *et al.*, 2008). Production of EPS by a subset of microorganisms entrained into sea ice might serve all entrapped within it, including common seawater species not adapted to life in ice, thereby limiting species-specific mortality.

The structure of these persistent winter sea ice communities is unique in resembling that of communities in autumn and winter seawater, measured in this study and reported by Alonso-Sáez and colleagues (2008) for our study site, rather than previously observed communities in spring and summer sea ice. For the first time, sea ice bacterial communities were observed (by both clone library and fingerprinting results) to be dominated by the common pelagic SAR11 clade *Alphaproteobacteria*, with only much smaller complements of the *Alphaproteobacteria* and *Flavobacteriales* well known from sea ice. While studies of later season sea ice have failed to detect *Archaea*, all of our winter ice (and seawater) samples contained them, with the dominant archaeon belonging to Marine Group I.1a *Crenarchaeota*. Clearly, these pelagic *Bacteria* and *Archaea* entrained into sea ice upon its formation and persisted through the winter. Our community structure results also indicate that microorganisms from other habitats, including terrestrial soil, riverine waters and marine sediment, entrain into sea ice and persist through the winter. Likely sources of the non-marine microorganisms include freshwater from the nearby Horton River, eroded soils from the Smoking Hills, and terrestrial organic matter from the Mackenzie River, the largest source of suspended particulates to the Beaufort Shelf (Macdonald *et al.*, 1998).

All of the dominant SAR11 phylotypes detected in our winter ice samples were highly similar to sequences from the coastal Beaufort Sea. Regarding the dominant SAR11 phylotypes detected in our winter ice samples, all were highly similar to sequences from the coastal Beaufort Sea (Galand *et al.*, 2008a), central Arctic Ocean (Bano and Hollibaugh, 2002; Malmstrom *et al.*, 2007), and Antarctic surface waters (Murray and Grzymski, 2007), even though absent from summer sea ice (Brown and Bowman, 2001; Brinkmeyer *et al.*, 2003) and highly productive autumn sea ice near Antarctica (Brinkmeyer *et al.*, 2003). The only cultured representative of the SAR11 clade is ‘*Candidatus* Pelagibacter ubique’, an obligately oligotrophic alphaproteobacterium distributed widely throughout the waters of the global ocean (Morris *et al.*, 2002). Although we detected two clades of SAR11 ITS sequences, no evidence was found to indicate ecotype differentiation (as suggested by García-Martínez and Rodríguez-Valera, 2000) between sea ice and seawater environments. Because sequences within the SAR11 clade made up a larger fraction of the late winter sea ice library (70%) than the under-ice seawater library (48%), SAR11 may nevertheless overwinter more successfully in ice than in seawater. This inference is consistent with quantitative FISH studies by Alonso-Sáez and colleagues (2008), who detected a seasonal decrease in the relative abundance of SAR11 in Franklin Bay surface waters from 36% of total DAPI counts at the time of freeze-up (December) to 18% by late winter (March).

The dominant archaeal phylotypes in our sea ice and seawater libraries, Marine Group I *Crenarchaeota* (Fig. 2, Table S4) have been identified from central Arctic seawater (Bano *et al.*, 2004), Beaufort shelf nepheloid layers and riverine particles (Galand *et al.*, 2006; 2008b;), and Antarctic seawater growing frazil ice (Delong *et al.*, 1994), yet were absent from summer sea ice (Brown and Bowman, 2001; Brinkmeyer *et al.*, 2003) and highly productive autumn sea ice near Antarctica (Brinkmeyer *et al.*, 2003). These phylotypes clustered with the ‘Marine’ Group I *Crenarchaeota* rather than the ‘Freshwater’ Group I which are prominent in the Mackenzie River (Galand *et al.*, 2008a,b;). The seasonally high relative abundance of the Marine Group I *Crenarchaeota* that we detected both by T-RFLP (45% of total signal intensity) and clone library sequencing (91% of sequences in both libraries) in Franklin Bay is consistent with FISH counts in surface waters at the same site, showing a winter high for this group (to 16% of DAPI counts) that decreased to undetectable levels by late summer (Alonso-Sáez *et al.*, 2008). This trend is also consistent with results from Antarctic waters showing that the relative abundance of Marine Group I *Crenarchaeota* was highest during winter and inversely correlated with algal biomass (chlorophyll *a*) on seasonal time scales (Murray *et al.*, 1998; Church *et al.*, 2003). The rarer Marine Group II *Euryarcheota* we detected were also closely related to phylotypes found prevalently in archaeal communities from the coastal Beaufort Sea (Galand *et al.*, 2006; 2008b;) and central Arctic Ocean (Bano *et al.*, 2004). Although *Archaea* were identified by Junge and colleagues (2004) in winter sea ice, their FISH probes were generic for the domain *Archaea* so no further taxonomic assignment was possible.

Bacterial sequences from several frequently cultured groups of copiotrophic sea ice bacteria were absent from our winter clone libraries. Neither the bacterial sea ice nor seawater library harboured sequences from cold-adapted genera of the *Oceanospirillales*, *Alteromonadales* or *Flavobacteriales* commonly cultured and cloned from sea ice, including *Colwellia*, *Glaciecola*, *Halomonas*, *Marinobacter*, *Pseudoalteromonas*, *Psychrobacter*, *Psychromonas* and *Shewanella* (Fig. S4, Table S3). Instead, the majority of gammaproteobacterial phylotypes were related to recently cultured oligotrophic seawater clades OM60 and OM182 (Cho and Giovannoni, 2004). The OM60 clade is prominent in Arctic seawater (Bano and Hollibaugh, 2002; Kellogg and Deming, 2009) while the OM182 clade is seasonally abundant in Antarctic surface waters (Murray *et al.*, 1998; Grzymski *et al.*, 2006; Murray and Grzymski, 2007). A representative from the OM60 clade was reported in Arctic pack ice (Brinkmeyer *et al.*, 2003), but no members of the OM182 clade have previously been identified from sea ice. Cultured isolates of sea ice *Alphaproteobacteria* generally cluster with marine *Roseobacter*, but only one of our phylotypes was most similar to a cultured representative (*Sulfitobacter* sp.). Others in the uncultured *Roseobacter* RCA cluster were detected infrequently in our libraries, consistent with their low abundance in seawater at our overwintering station (< 5%; Alonso-Sáez *et al.*, 2008). *Bacteroidetes* are well known from sea ice and often make up a sizable fraction of the community in polar seawater (Bano and Hollibaugh, 2002; Wells and Deming, 2003; Malmstrom *et al.*, 2007), including at our overwintering site (Alonso-Sáez *et al.*, 2008), yet made up only a small fraction of the bacterial clones in our winter sea ice library. The implication is that these common sea ice bacteria only become dominant in sea ice after the winter season has passed.

*Polaribacter*, a genus of *Bacteroidetes* appearing in the ARISA analysis, provided a notable exception to the lack of representation by cultivated isolates in the winter sea ice we sampled (Table 1 and Table S1). *Polaribacter* spp., known for their production of gas vacuoles, have been cultured and identified as abundant *Bacteroidetes* from both Arctic and Antarctic sea ice (Gosink *et al.*, 1998; Brown and Bowman, 2001; Junge *et al.*, 2002; Brinkmeyer *et al.*, 2003; Auman *et al.*, 2006). The recent genome sequence of Antarctic seawater isolate *Polaribacter irgensii* strain 23-P (GenBank accession: AAOG00000000) may help elucidate their adaptations to sea ice, just as the genome sequence analysis of *Polaribacter* MED152 has revealed their adaptations to sunlit surface waters (e.g. rhodopsins; González *et al.*, 2008). In this study, preferential entrainment of *Polaribacter* spp. into sea ice was inconclusive, because they were also detected in under-ice seawater by both cloning and ARISA, but the high intra-specific ITS variability we observed may prove useful in future tests of ecotype differentiation by these successful sea ice colonizing bacteria.

Overall, the dominant presence of common seawater microorganisms and the absence of many known sea ice microorganisms in the Arctic winter ice we studied indicate that species-specific mortality during the winter was rare. The degree of similarity between winter ice and seawater communities also implies that selection during the freezing process must have been relatively minor. The distinctive nature of well-known microbial communities in sea ice of the warmer biologically productive seasons must not be predetermined by selective survival of community members exposed to freezing and severe winter conditions, but rather as a result of competitive outgrowth by copiotrophs that overwinter below detection limit or arrive as immigrants once the warming ice becomes permeable.

## Experimental procedures

### Ice core and seawater sampling

Sampling location and procedures have been reported in detail by Collins and colleagues (2008), along with measurements of air and ice temperature, bulk ice and brine salinity, brine volume fraction, and content of total particulate matter, bacterial abundance and pEPS in the ice. Briefly, each week from 10 January (calendar day 10) to 28 March 2004 (day 88), three ice cores were drilled from a designated field of landfast first-year sea ice in Franklin Bay, Northwest Territories, Canada (at 70.0°N, 126.3°W; 16 km from the mouth of the Horton River) without reaching seawater to capture 10 cm depth horizons centred at 25, 45 and 65 cm from the ice surface and designated horizons I, II and III respectively. Freezing dates for horizons I and II were predicted to be 9–18 November and 26 November–5 December, respectively, while horizon III was observed to freeze from 14 to 20 December 2003.

The ice sections were cut aseptically in the field, placed into sterile Whirl-Pak bags and transported in an insulated cooler to a shipboard cold room set at 0°C, where they were processed within 24 h. To protect against osmotic shock and possible cell lysis, each section (after mechanical crushing) was melted into 0.22 µm filtered artificial brine solution (prepared as in Collins *et al.*, 2008) at 0°C, using an ice : brine volume ratio of 1:2. After subsampling for other variables (Collins *et al.*, 2008), the remainder of each melted sample was gently filtered onto a 47 mm diameter 0.22-µm-nitrocellulose filter (Millipore) and stored at −80°C for later DNA extraction.

Under-ice seawater samples were collected on calendar days 35 and 88 by lowering a hand-held 2 l Niskin bottle through a hole in the ice to the base of the ice sheet. Designated 35-SW and 88-SW, the seawater samples, each with a salinity of 30, were returned to the ship, filtered immediately, and the filters stored at −80°C, as for fully melted sea ice samples.

### DNA extraction

Within 2 years of collection, each filter was removed from storage at −80°C and cut into small fragments with sterilized scissors. For horizons II and III, all three filters (one from each of the three ice cores) from each sampling day were combined in a single tube. In horizon I, due to low DNA yields, six filters – three from each of two sampling days in adjacent weeks – were combined in a single tube, i.e. from the following pairs of calendar sampling days: 10 and 17, 24 and 31, 60 and 67, and 74 and 81. Each tube then received, per filter, 800 µl of STE buffer (100 mM NaCl, 10 mM Tris-HCl, 1 mM EDTA, pH 8.0) and 40 µl of 20% SDS. After incubation at 65°C for 20 min, each tube was vortexed, then centrifuged at 1400 *g* for 15 min. The supernate was transferred to a Centricon YM-100 centrifugal filtration device (Millipore) to concentrate and de-salt the genomic DNA, according to the manufacturer's recommendations. The recovered volume was increased to 600 µl with TE buffer before three rounds of phenol/chloroform extraction, followed by ethanol precipitation and re-suspension in 50 µl of TE buffer. Total DNA concentration was measured in a SpectraMaxM2 plate reader (Molecular Devices) using PicoGreen fluorescence (Invitrogen) according to the manufacturer's recommendations. Recovery of genomic DNA was < 1–33% based on cell counts (Collins *et al.*, 2008), assuming 2.5 fg of DNA per bacterium (Button and Robertson, 2001). No attempt was made to separate the DNA of viable cells from that of dead cells.

### Community fingerprinting

Bacterial DNA fragments were PCR-amplified for ARISA using fluorescently labelled (6-HEX) forward primer Uni1392F, and unlabelled reverse primer R23S-125R (sequences located in Table S5). Amplified bacterial DNA was pooled from two PCR amplifications, each containing 3–117 ng of total DNA. Partial archaeal 16S rRNA genes were PCR-amplified for T-RFLP using fluorescently labelled (6-FAM) forward primer Arch109F, and unlabelled reverse primer Arch915R. Amplified archaeal DNA from four PCR amplifications was pooled, then digested with restriction enzyme HpyCH4III at 37°C for 6 h, which was determined empirically to enable complete digestion without overdigestion. PCR amplifications using archaeal primers were generally more robust than with bacterial primers, even though archaeal abundance was likely only a few per cent of the bacterial abundance (Junge *et al.*, 2004), a phenomenon which has also been observed in a highly saline Arctic spring system (T. Niederberger, pers. comm.). All DNA fragments were analysed on a MegaBACE1000 capillary gel electrophoresis instrument (Molecular Dynamics).

Electropherograms were analysed using DAx analysis software (v8.0, Van Mierlo Software Consultancy). A low-pass Fourier transform was applied to ARISA electropherograms to reduce noise and increase peak calling efficiency. Heights of saturated peaks in several T-RFLP electropherograms were estimated by fitting a Gaussian function to the non-saturated points, using the open-source statistical package R (R Development Core Team, 2008). For both ARISA and T-RFLP, a peak was called if its height was > 5× the baseline root-mean-square noise level (< 1.0% of the cumulative peak height for each profile). Profiles with cumulative peak heights less than 1 × 10^4^ RFUs (ARISA) or 8 × 10^4^ RFUs (T-RFLP) were removed from the analysis. The peaks in the remaining samples were binned using in-house software (http://rocaplab.ocean.washington.edu/cgi/dakster/index.html) and distance cut-offs of 1 bp for fragment lengths of 70–700 bp, 2 bp for 700–1200 bp and 4 bp for > 1200 bp. Each bin, representing a 16S-ITS-23S ribosomal DNA fragment (ARISA) or terminal restriction fragment (T-RFLP), was designated an OTU. The cumulative peaks heights of each OTU were used as gross measures of relative abundance to compare fingerprinting with clone libraries. A presence/absence matrix containing OTUs with at least one peak height greater than 1.0% (ARISA) or 0.25% (T-RFLP) of the sample's cumulative peak height was used for calculation of richness and pairwise similarity, and non-parametric multivariate analyses, performed with PRIMER v6 (Clarke and Gorley, 2006). Pairwise distances were calculated as Sørensen's similarity coefficient (C_s = 2C/(A + B), where A and B are the number of OTUs in each of two samples, and C is the number of shared OTUs between the two samples; Hughes *et al.*, 2001); samples were then clustered by Group Average and the significance of each cluster was calculated by SIMPROF at the 95% confidence level. Pearson correlation coefficients for Sørensen's similarity with date were calculated in R, as were partial Pearson correlation coefficients for richness with date controlling for the cumulative peak height of each profile. A correlation function in the open-source plotting program Qtiplot was used to calculate the point of maximum covariance between ARISA RFUs and clone library phylotype frequencies to correct for different running rates of fluorescent labels 6-HEX and ROX (used for the ARISA ladder), resulting in an adjustment of ARISA OTU lengths by −7 bp, near the −5.5 bp difference determined by Hahn and colleagues (2001).

### Clone library construction and sequencing

Four clone libraries were constructed for phylogenetic analysis, using the ribosomal RNA operon, from two samples: under-ice seawater from day 35, used to create libraries FB04bw and FB04aw; and winter sea ice from horizon I (combined days 74 and 81), used to create libraries FB04bi and FB04ai, where FB04 = Franklin Bay 2004, b = *Bacteria*, a = *Archaea*, w = water and i = ice. Bacterial 16S-ITS-23S ribosomal DNA was amplified using primer pair Uni515F/R23S-125R (Table S5). Archaeal 16S ribosomal DNA was amplified using primer pair Arch21F/Arch958R. Four separate amplifications were performed for each sample and each was reconditioned using 1/10 of the PCR product as template for an additional four cycles with fresh reaction mix (Thompson *et al.*, 2002). The pooled reconditioned PCR product was cloned using the TOPO-TA Cloning Kit for Sequencing (Invitrogen) according to the manufacturer's recommendations. Bacterial clone libraries were subjected to dye-termination sequencing at the High-Throughput Genomics Unit (HTGU), Department of Genome Sciences, University of Washington. Archaeal clone libraries were sequenced on a MegaBACE1000 capillary gel electrophoresis instrument. Bi-directional double-stranded sequences were obtained for > 900 bp of the bacterial and archaeal 16S rRNA genes and the complete ITS region for *Bacteria*. Sequencher software (v4.6, Gene Codes Corp.) was used to call bases and construct contigs which were checked and edited manually as necessary. Most of the bacterial sequences included the 16S–23S ITS region, which we excluded from phylogenetic analyses but from which we calculated predicted ARISA fragment lengths and defined subtypes of 16S rRNA gene phylotypes. Seventeen sequences related to *Stenotrophomonas maltophila* were removed from the bacterial sea ice library as probable contaminants. ARISA fragment lengths predicted from these sequences (777 and 779 bp) overlapped with those predicted from other clone library sequences, but these fragments were observed only rarely in the ARISA data set. Bacterial sequences were deposited in GenBank with Accession No. EU836892–EU837057 and FJ753995–FB754002; archaeal sequences: EU486859–EU486955; and eukaryotic sequences: FJ753982–FJ753994.

### Phylogenetic analysis

Small-subunit ribosomal rRNA gene sequences were aligned using the NAST aligner (Desantis *et al.*, 2006) at Greengenes (http://greengenes.lbl.gov). All sequences were checked for chimeras with Bellerophon (Huber *et al.*, 2004) and Mallard (Ashelford *et al.*, 2006); none were detected. Sequence alignments were imported into ARB (Ludwig *et al.*, 2004) and edited manually as necessary. Bootstrapped phylogenetic trees were constructed in paup* v4.10beta (Swofford, 2003). ModelTest was used to determine the optimal nucleotide substitution model for maximum likelihood tree construction (Posada and Crandall, 1998). In ARB, partial sequences were added to the tree by parsimony and assigned phylotypes based on their location within the tree. Distance matrices calculated in paup* using the Tamura and Nei (1993) model of nucleotide substitution were used with WebLIBSHUFF (Schloss *et al.*, 2004) to compare clone libraries statistically, and with dotur (Schloss and Handelsman, 2005) to define phylotypes and calculate estimates of species richness and diversity using the Chao1 and Shannon indices respectively. Phylotypes were defined by > 98% similarity for bacterial sequences and > 99% for archaeal sequences. Subtypes were designated if multiple fingerprinting fragments were predicted within any single phylotype. The archaeal sequences were subsequently used to aid the choice of restriction enzymes for community profiling by T-RFLP (Collins and Rocap, 2007).

## Supporting information

**Fig. S1.** Bacterial abundance (A–C) from Collins and colleagues (2008), and the community richness (D–F) of *Bacteria* (×, 98% similarity cut-off) and *Archaea* (□, 99% similarity cut-off) in Arctic winter sea ice horizons I–III, representing depths of 25, 45 and 65 cm below the ice surface (top, middle and bottom panels, respectively). Dashed lines in (A) and (B) indicate robust linear best fit lines in horizons with statistically significant decreases (*P* < 0.05) in cell abundance over time. Pearson correlation coefficients of richness over time were not significant for either community in any horizon.

**Fig. S2.** Map of the vicinity of Franklin Bay, Northwest Territories, Canada, indicating Franklin Bay (arrows), the primary sampling site at the CCGS *Amundsen* overwintering station (o) and nearby Smoking Hills (x).

**Fig. S3.** Phylogenetic tree of alphaproteobacterial and betaproteobacterial 16S rRNA gene sequences. Tree topology was defined by the consensus of 1000 maximum parsimony bootstrap replications utilizing 291 parsimony-informative nucleotides. Branch lengths were defined by Tamura-Nei distances calculated from 558 hypervariable-masked nucleotides. Node values indicate percentage of 10 000 distance, 1000 maximum parsimony and 100 maximum likelihood bootstrap replications respectively; only bootstrap values greater than 50% are shown. One sequence from each phylotype (defined by > 98% similarity) from each library in this study is shown in bold, followed in parentheses by the phylotype and number of sequences from each library within that phylotype.

**Fig. S4.** Phylogenetic tree of gammaproteobacterial 16S rRNA gene sequences. Tree topology was defined by the consensus of 1000 maximum parsimony bootstrap replications utilizing 240 parsimony-informative nucleotides. Branch lengths were defined by Tamura-Nei distances calculated from 570 hypervariable-masked nucleotides. Other features as in Fig. S3.

**Table S1.** Bacterial ARISA OTUs in Franklin Bay (FB) sea ice horizons I–III (representing depths of 25, 45 and 65 cm below the ice surface) and under-ice seawater (SW). Each horizon is ordered by calendar day (left to right). Shown are OTUs that matched the predicted fragment length from a clone library sequence and OTUs present in at least 70% of the sea ice samples, excepting OTUs present in Table 1. A black dot indicates the presence of a peak; alternating grey and white rows are used for clarity. ‘% height’ indicates the percentage of the total global peak height in each OTU.

**Table S2.** Archaeal T-RFLP OTUs in Franklin Bay (FB) sea ice horizons I–III (representing depths of 25, 45 and 65 cm below the ice surface), and under-ice seawater (SW). Each horizon is ordered by calendar day (left to right). Shown are OTUs present in at least 50% of the sea ice samples, and those with at least one peak height greater than 0.25% of the sample’s cumulative peak height, excepting OTUs present in Table 2. A black dot indicates the presence of a peak; alternating grey and white rows are used for clarity. ‘% height’ indicates the percentage of the total global peak height in each OTU. Best sequence database matches are demarcated by origin: ^†^this study; ^‡^ Galand and colleagues (2006); and ^§^GenBank. MGI, Marine Group I *Crenarchaeota*; MGII, Marine Group II *Euryarchaeota*; LDS and RC-V refer to clades of uncultured *Euryarchaeota*.

**Table S3.** Bacterial phylotypes (defined by > 98% 16S rRNA gene similarity) in clone libraries from late winter sea ice (FB04bi) and under-ice seawater (FB04bw). Subtypes were designated by predicted ARISA lengths, calculated for primer pair Uni1392F and R23S-125R. ‘Unknown’ indicates partial sequence for which ARISA length could not be calculated.

**Table S4.** Archaeal phylotypes (defined by > 99% 16S rRNA gene similarity) in clone libraries from late winter sea ice (FB04ai) and under-ice seawater (FB04aw). Subtypes were designated by predicted TRF lengths, calculated for primer/enzyme combination Arch109F/HpyCH4III. ‘Unknown’ indicates partial sequence for which TRF length could not be calculated.

**Table S5.** Primers used in this study. Universal name as described by Alm and colleagues (1996).
