# Supplementary material for: Persistence of bacterial and archaeal communities in sea ice through an Arctic winter
Source: Environ Microbiol. 2010 Jul;12(7):1828–41. doi: 10.1111/j.1462-2920.2010.02179.x (PMC2916213; doi:10.1111/j.1462-2920.2010.02179.x)
Supplement: Supplementary file 1 [file emi0012-1828-SD1.pdf]

Supplementary Materials

Persistence of Bacterial and Archaeal communities in sea ice through an Arctic winter

R. Eric Collins*, Gabrielle Rocap, and Jody W. Deming
* corresponding author: R. Eric Collins

School of Oceanography
Box 357940
1503 NE Boat St.
University of Washington
Seattle, WA 98195 USA
Tel 1-206-221-5755
Fax 1-206-543-0275

Submitted to Environmental Microbiology
Running title: Bacteria and Archaea in Arctic winter sea ice

December 27, 2009

Supplementary Table 1: Bacterial ARISA OTUs in Franklin Bay (FB) sea ice horizons I–III (representing depths of 25, 45, and 65 cm below the ice surface) and under-ice seawater (SW). Each horizon is ordered by calendar day (left to right). Shown are OTUs that matched the predicted fragment length from a clone library sequence and OTUs present in at least 70% of the sea ice samples, excepting OTUs present in Table 1. A black dot indicates the presence of a peak; alternating gray and white rows are used for clarity. '% height' indicates the percentage of the total global peak height in each OTU.

	FB I			FB II							FB III	FB SW	%	Phylotype of best clone
	24	60	74	24	31	38	53	60	67	74	31	88	height	library match
672	•	•	•	•	•		•	•	•	•	•	•	3.5	—
680	•	•	•	•		•	•	•	•	•	•	•	3.3	SAR11: B1-a, B2, B4, B5-b, B7, B8; OM182: B17-a
816	•	•	•	•		•	•	•	•	•	•		1.4	Alphaproteobacteria: B12; Methylophilus: B24; Polaribacter: B28-b
839	•	•	•		•	•		•	•	•	•	•	1.4	Methylophilus: B23
820	•	•	•	•	•			•	•	•	•		1.0	Chromatiales: B22
734	•	•	•		•			•	•	•	•		0.8	—
595	•	•	•	•	•	•		•		•	•	•	0.7	Cryomorphaceae: B32
784	•							•		•			0.7	Polaribacter: B28-a
1100	•	•	•		•			•	•	•			0.6	OM182: B18-b
781			•							•			0.6	Janthinobacterium: B25
544	•	•	•		•					•		•	0.5	Cryomorphaceae: B29
777	•		•					•		•	•		0.5	OM182: B19
664	•						•		•			•	0.5	SAR11: B1-b, B3-b, B5-a, B6
801			•							•			0.5	Proteobacteria: B27; Actinobacteria: B43
551	•		•					•			•	•	0.4	Cryomorphaceae: B29, B31
600	•	•	•		•	•				•			0.4	Cryomorphaceae: B32
531		•	•					•	•	•			0.2	Cryomorphaceae: B34
548												•	0.2	Cryomorphaceae: B29, B31
779	•		•										0.1	Janthinobacterium: B25
513		•											0.1	Saprospira: B40
775	•												0.1	Formosa: B38
623								•	•		•		0.1	Marine Group A: B44
850								•			•		0.1	Polaribacter: B28-c
697			•										0.1	Alphaproteobacteria: B13-a; OM182: B17-b
825								•					0.1	Chromatiales: B22
847								•				•	0.1	Deltaproteobacteria: B26; Polaribacter: B28-c
629												•	0.1	Marine Group A: B44
676			•										< 0.1	SAR11: B1-a
769										•			< 0.1	Flavobacteriaceae: B35
1016	•												< 0.1	Verrucomicrobia: B41

Supplementary Table 2: Archaeal T-RFLP OTUs in Franklin Bay (FB) sea ice horizons I-III (representing depths of 25, 45, and 65 cm below the ice surface), and under-ice seawater (SW). Each horizon is ordered by calendar day (left to right). Shown are OTUs present in at least 50% of the sea ice samples, and those with at least one peak height greater than 0.25% of the sample's cumulative peak height, excepting OTUs present in Table 2. A black dot indicates the presence of a peak; alternating gray and white rows are used for clarity. '% height' indicates the percentage of the total global peak height in each OTU. Best sequence database matches are demarcated by origin: †, this study; ‡, Galand et al. (2006); and §, GenBank. MGI, Marine Group I Crenarchaeota; MGII, Marine Group II Euryarchaeota; LDS and RC-V refer to clades of uncultured Euryarchaeota.

	FB I			FB II									FB III									FB SW		%	Taxonomic groups of
	24	60	74	17	24	31	38	53	60	67	74	81	10	17	24	31	53	60	67	74	81	35	88	height	best database matches
219																	●					●		1.0	—
94									●		●	●							●			●	●	0.8	—
91					●														●					0.8	MGII.b†‡, MGII.a†‡, RC-V‡
513					●														●					0.5	MGI.3a‡
524			●	●	●	●	●	●	●	●	●	●	●		●	●	●	●	●	●	●	●	●	0.4	Freshwater Group I.1a‡
87		●				●		●	●	●	●	●	●			●	●	●			●		●	0.4	RC-V‡
531				●					●	●			●		●	●	●	●		●	●	●		0.4	—
529				●			●		●						●	●	●	●	●					0.4	—
222			●		●	●		●	●			●	●			●		●	●		●	●	●	0.4	RC-V‡, LDS‡
321											●								●			●	●	0.3	—
761	●	●	●	●	●	●	●	●	●	●	●	●				●	●	●	●		●	●	●	0.3	—
282		●	●				●	●	●	●	●					●			●			●	●	0.3	Methanomicrobiales‡§
461											●	●									●		●	0.3	—
380		●	●	●	●		●		●	●	●	●	●			●	●	●	●	●	●	●	●	0.2	—
325					●														●			●		0.2	—
516					●														●				●	0.2	—
458																	●					●		0.2	—
301																	●					●		0.2	Desulfurococcales§
272		●	●	●	●		●		●	●	●	●	●		●	●	●	●	●		●	●	●	0.2	—
322					●		●					●					●	●	●		●			0.2	—
515			●	●	●		●		●															0.2	MGI.3a‡
498		●	●	●	●		●	●	●	●	●	●	●		●	●	●	●	●	●	●	●		0.1	MGI.1c†‡
455		●			●	●	●	●	●	●	●	●	●			●	●	●	●	●	●	●	●	0.1	—
609			●	●	●	●	●					●		●	●	●	●	●	●		●	●		0.1	*Methanococcus*§,*Picrophilus*§
477				●	●			●	●				●		●	●	●	●	●	●	●	●		0.1	Methanobacteria§
567																				●				0.1	—
287		●	●	●	●	●	●		●	●	●	●	●		●	●	●	●	●	●	●	●	●	0.1	MGI.3c‡
157		●		●	●	●			●	●		●	●		●	●	●	●	●	●	●	●		0.1	—
499				●	●	●	●	●	●	●	●	●	●			●	●	●			●		●	0.1	MGI.1c†‡
132		●			●			●	●	●		●	●			●	●	●	●		●	●	●	0.1	MGI.1a†
137		●	●		●	●	●	●	●	●	●	●				●		●				●		0.1	—
228		●						●	●													●		0.1	*Thermoplasma*§
247																	●					●		0.1	—
231													●											0.1	—
197																				●				< 0.1	—

Supplementary Table 3: Bacterial phylotypes (defined by >98% 16S rRNA gene similarity) in clone libraries from late winter sea ice (FB04bi) and under-ice seawater (FB04bw). Subtypes were designated by predicted ARISA lengths, calculated for primer pair Uni1392F and R23S-125R. 'Unknown' indicates partial sequence for which ARISA length could not be calculated.

Taxonomy	Phylotype	Sea ice clones	Seawater clones	ARISA length
	**Proteobacteria**: Alphaproteobacteria			
SAR11	B1-a	41	16	678–683
	B1-b	3	3	661–663, 665–666
	B1-unknown	1	—	—
SAR11	B2	14	2	680–682
SAR11	B3-a	2	—	682
	B3-b	3	—	664
SAR11	B4	3	—	680
SAR11	B5-a	1	—	663
	B5-b	2	—	679
SAR11	B6	2	—	663
SAR11	B7	2	—	681
SAR11	B8	1	—	681
SAR11	B9	1	—	662
SAR11	B10	—	1	662
Sulfitobacter	B11-unknown	1	—	—
Roseobacter cluster	B12	2	—	813
Unclassified	B13-a	1	—	699
	B13-b	1	—	702
Unclassified polar	B14	—	1	1051
Roseobacter cluster	B15-unknown	—	1	—
Roseobacter cluster	B16-unknown	—	1	—
	**Proteobacteria**: Gammaproteobacteria			
OM182	B17-a	2	1	681–682
	B17-b	1	—	695
OM182	B18-a	2	—	916–917
	B18-b	2	—	1096
OM182	B19	2	—	777
OM182	B20	1	—	702
OM60	B21-a	1	—	979
	B21-unknown	—	1	—
Chromatiales	B22	1	—	820
	**Proteobacteria**: Betaproteobacteria			
Methylophilus	B23	—	1	837
Methylophilus	B24	1	—	816
Janthinobacterium	B25	1	—	779
	**Proteobacteria**: Deltaproteobacteria			
Unclassified	B26	1	—	844
	**Proteobacteria**: Unclassified			
Unclassified	B27	1	—	796
	**Bacteroidetes**: Flavobacteria			
Polaribacter	B28-a	2	—	786

(cont.)

Taxonomy	Phylotype	Sea ice clones	Seawater clones	ARISA length
	B28-b	—	1	817
	B28-c	1	—	845
	B28-d	1	—	856
Cryomorphacaea	B29	—	4	548–549
Cryomorphaceae	B30	—	1	709
Cryomorphaceae	B31	—	1	549
Cryomorphaceae	B32	—	1	596
Cryomorphaceae	B33	—	1	719
Cryomorphaceae	B34	—	1	532
Flavobacteriaceae	B35	1	1	770–771
Flavobacteriaceae	B36	1	—	—
Cellulophaga	B37	—	1	1085
Formosa	B38	—	1	776
**Bacteroidetes**: Sphingobacteria				
Unclassified	B39	2	—	895
Saprospira	B40	—	3	514
**Verrucomicrobia**				
Verrucomicrobaceae	B41	1	—	1012
**High G+C Gram Positive**: Actinobacteria				
Cryobacterium	B42	3	—	661–662
Unclassified	B43	—	1	796
**'Marine Group A'**				
Unclassified	B44	—	1	624

Supplementary Table 4: Archaeal phylotypes (defined by >99% 16S rRNA gene similarity) in clone libraries from late winter sea ice (FB04ai) and under-ice seawater (FB04aw). Subtypes were designated by predicted TRF lengths, calculated for primer/enzyme combination Arch109F/HpyCH4III. 'Unknown' indicates partial sequence for which TRF length could not be calculated.

Taxonomy	Phylotype	Sea ice clones	Seawater clones	TRF length
		**Crenarchaeota: Marine Group I**		
Cluster 1a	A3-a	34	31	527–528
	A3-b	1	—	132
	A3-unknown	8	4	—
Cluster 1a	A4-a	1	1	528
	A4-unknown	1	2	—
Cluster 1a	A5	1	1	528
Cluster 1a	A6	—	1	528
Cluster 1c	A7	—	1	499
		**Euryarchaeota: Group II.b**		
Cluster 7	A1-a	4	2	93
	A1-unknown	—	1	—
Cluster 5	A2	2	1	93

Supplementary Table 5: Primers used in this study. Universal name as described by Alm et al. (1996).

Common name	Universal name	Sequence (5'–3')	Usage	Reference
Arch21F	S-D-Arch-0002-a-S-20	TTCCGGTTGATCCYGCCGGA	Archaeal cloning	DeLong (1992)
Arch109F	S-D-Arch-0109-a-S-17	ACKGCTCAGTAACACGT	T-RFLP	Grosskopf et al. (1998)
Arch915R	S-D-Arch-0915-a-A-20	GTGCTCCCCCGCCAATTCCT	T-RFLP	Stahl and Amann (1991)
Arch958R	S-D-Arch-0958-a-A-19	YCCGGCGTTGAMTCCAATT	Archaeal cloning	DeLong (1992)
Uni515F	S-*-Uni-0515-a-S-19	GTGCCAGCMGCCGCGGTAA	Archaeal sequencing, Bacterial cloning	Lane (1991)
Uni515R19	S-*-Uni-0515-a-A-19	TTACCGCGGCKGCTGGCAC	Archaeal sequencing	Lane (1991)
Uni1392F	S-*-Uni-1392-a-S-15	GYACACACCGCCCGT	Bacterial sequencing, ARISA	Hewson and Fuhrman (2004)
Uni1392R	S-*-Uni-1392-a-A-15	ACGGGCGGTGTGTRC	Bacterial sequencing	Field et al. (1988)
R23S-125R	L-D-Bact-0125-a-A-15	GGTTBYCCCATTCRG	Bacterial cloning, ARISA	modification of Hunt et al. (2006)
M13F	—	GTAAAACGACGGCCAG	Archaeal and Bacterial sequencing	Invitrogen
M13R	—	CAGGAAACAGCTATGAC	Archaeal and Bacterial sequencing	Invitrogen

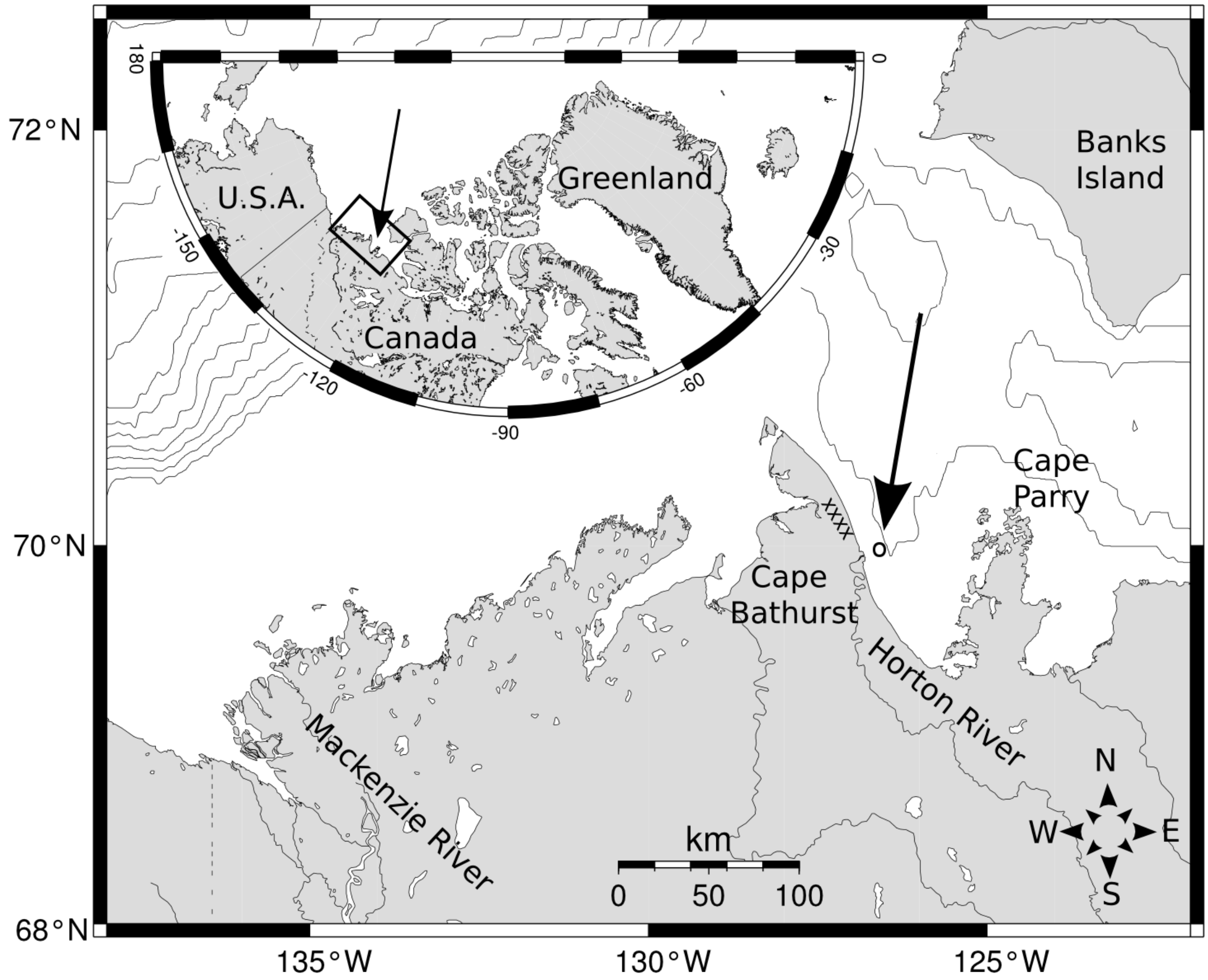

Supplementary Figure 1: Map of the vicinity of Franklin Bay, Northwest Territories, Canada, indicating Franklin Bay (arrows), the primary sampling site at the CCGS *Amundsen* overwintering station (∘) and nearby Smoking Hills (x).

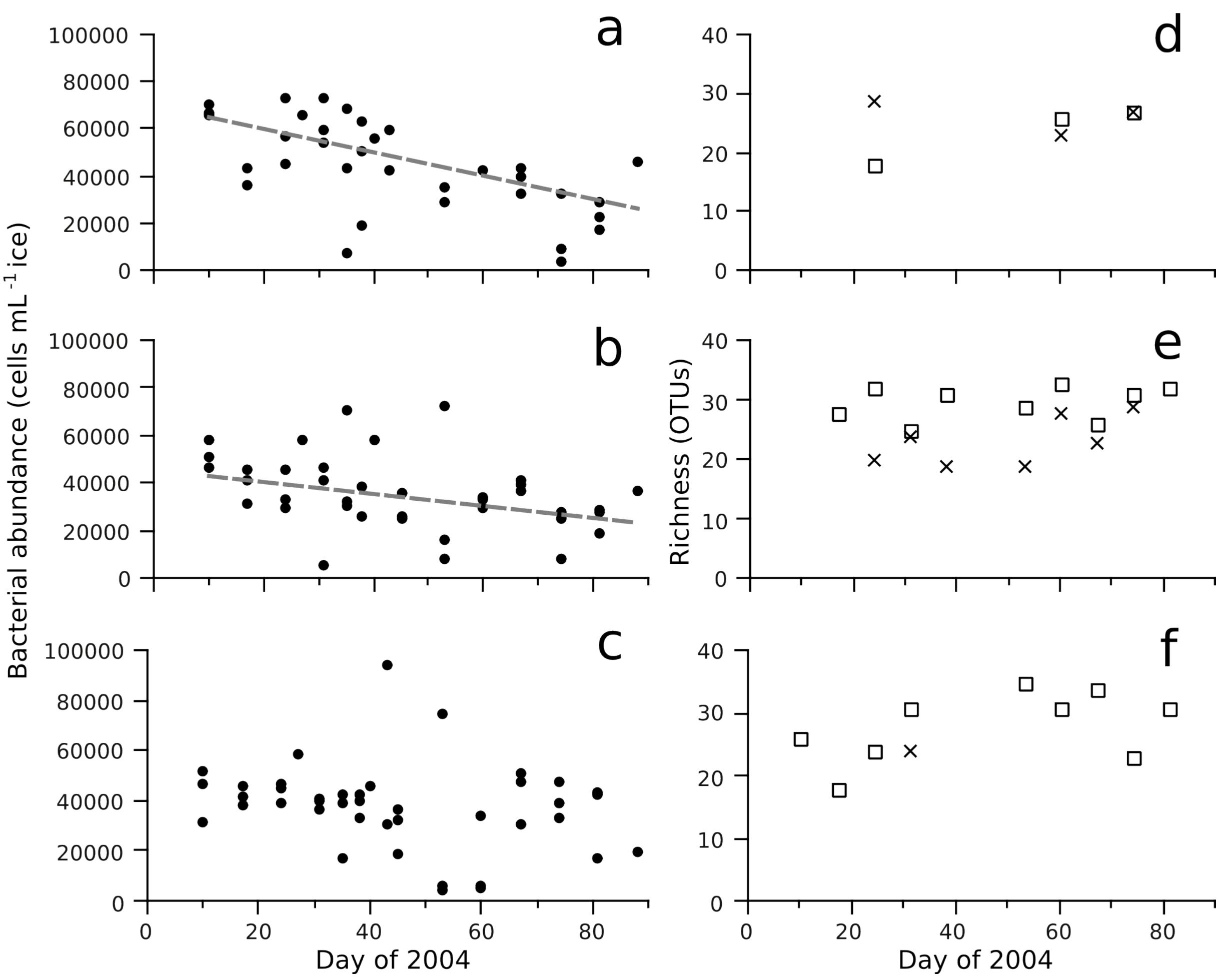

Supplementary Figure 2: Bacterial abundance (a–c) from Collins et al. (2008), and the community richness (d–f) of Bacteria (×, 98% similarity cutoff) and Archaea (□, 99% similarity cutoff) in Arctic winter sea ice horizons I–III, representing depths of 25, 45, and 65 cm below the ice surface (top, middle, and bottom panels, respectively). Dashed lines in (a) and (b) indicate robust linear best fit lines in horizons with statistically significant decreases ($p < 0.05$) in cell abundance over time. Pearson correlation coefficients of richness over time were not significant for either community in any horizon.

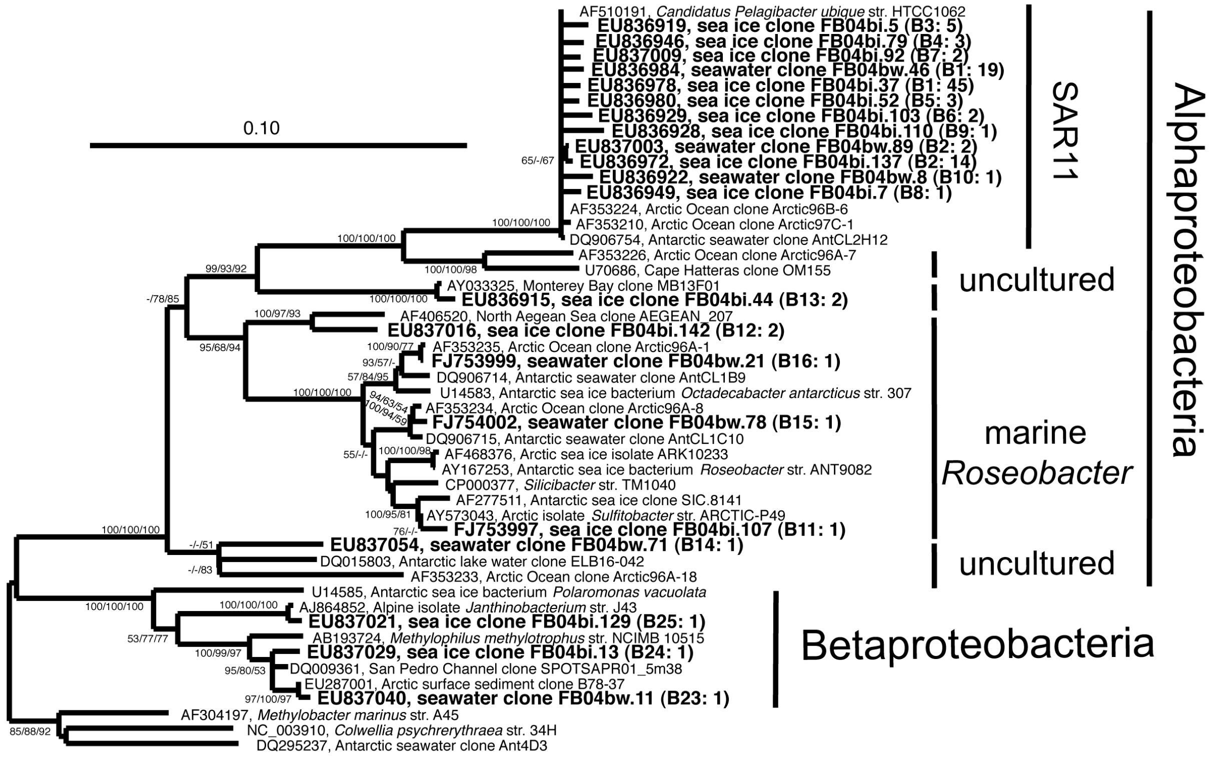

Supplementary Figure 3: Phylogenetic tree of alphaproteobacterial and betaproteobacterial 16S rRNA gene sequences. Tree topology was defined by the consensus of 1000 maximum parsimony bootstrap replications utilizing 291 parsimony-informative nucleotides. Branch lengths were defined by Tamura-Nei distances calculated from 558 hypervariable-masked nucleotides. Node values indicate percentage of 10000 distance, 1000 maximum parsimony, and 100 maximum likelihood bootstrap replications, respectively; only bootstrap values greater than 50% are shown. One sequence from each phylotype (defined by >98% similarity) from each library in this study is shown in bold, followed in parentheses by the phylotype and number of sequences from each library within that phylotype.

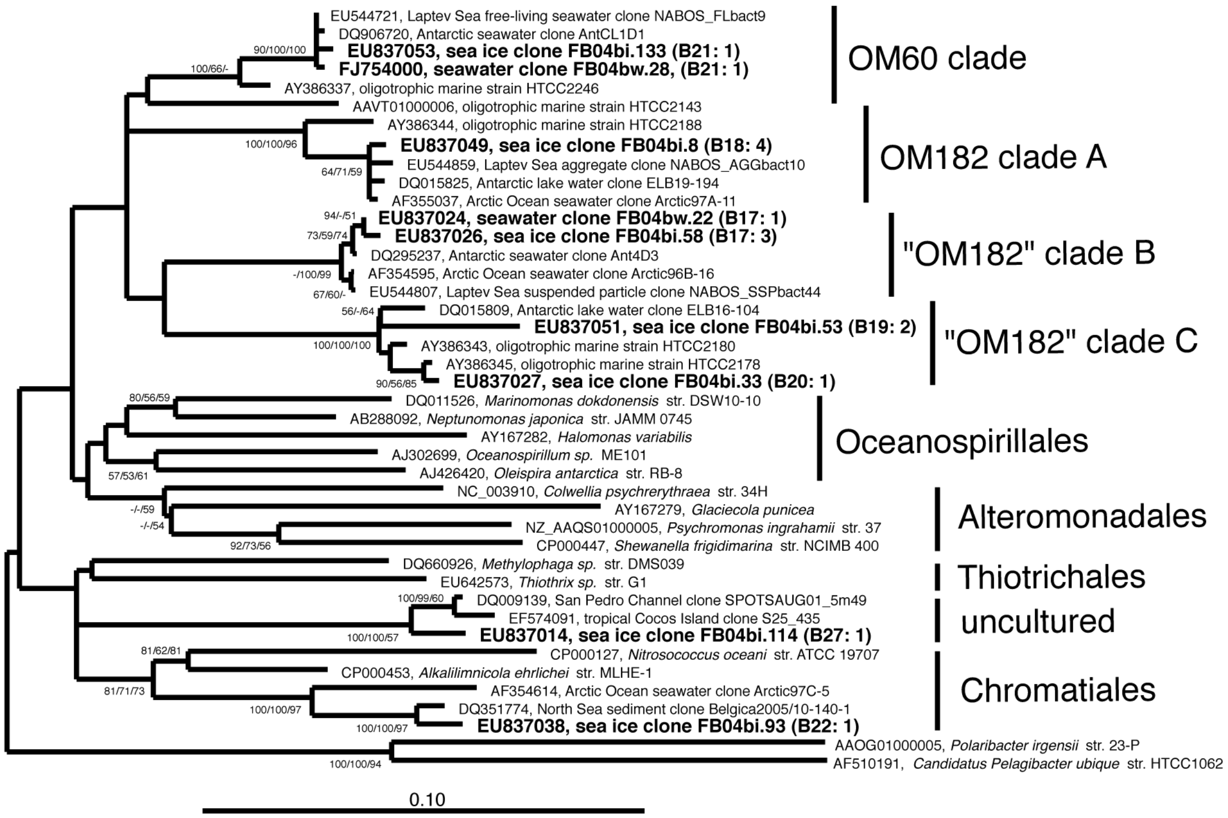

Supplementary Figure 4: Phylogenetic tree of gammaproteobacterial 16S rRNA gene sequences. Tree topology was defined by the consensus of 1000 maximum parsimony bootstrap replications utilizing 240 parsimony-informative nucleotides. Branch lengths were defined by Tamura-Nei distances calculated from 570 hypervariable-masked nucleotides. Other features as in Fig. S3.

References

Alm, E.W., Oerther, D.B., Larsen, N., Stahl, D.A., and Raskin, L. (1996) The oligonucleotide probe database. *Appl Environ Microbiol* 62:3557–3559.

Collins, R.E., Carpenter, S.D., and Deming, J.W. (2008) Spatial heterogeneity and temporal dynamics of particles, bacteria, and pEPS in Arctic winter sea ice. *J Mar Sys* 74:902–917.

DeLong, E.F. (1992) Archaea in coastal marine environments. *Proc Natl Acad Sci U S A* 89:5685–5689.

Field, K.G., Olsen, G.J., Lane, D.J., Giovannoni, S.J., Ghiselin, M.T., Raff, E.C., et al. (1988) Molecular phylogeny of the animal kingdrom. *Science* 239:748–753.

Galand, P.E., Lovejoy, C., and Vincent, W.F. (2006) Remarkably diverse and contrasting archaeal communities in a large Arctic river and the coastal Arctic Ocean. *Aquat Microb Ecol* 44:115–126.

Grosskopf, R., Janssen, P.H., and Liesack, W. (1998) Diversity and structure of the methanogenic community in anoxic rice paddy soil microcosms as examined by cultivation and direct 16S rRNA gene sequence retrieval. *Appl Environ Microbiol* 64:960–969.

Hewson, I. and Fuhrman, J.A. (2004) Richness and diversity of bacterioplankton species along an estuarine gradient in Moreton Bay, Australia. *Appl Environ Microbiol* 70:3425–3433.

Hunt, D.E., Klepac-Ceraj, V., Acinas, S.G., Gautier, C., Bertilsson, S., and Polz, M.F. (2006) Evaluation of 23S rRNA PCR primers for use in phylogenetic studies of bacterial diversity. *Appl Environ Microbiol* 72:2221–2225.

Lane, D.J. (1991) 16S/23S rRNA sequencing. In *Nucleic Acid Techniques in Bacterial Systematics* (Stackebrandt, E. and Goodfellow, M., eds.). New York: Wiley, 115–175.

Stahl, D.A. and Amann, R. (1991) Development and application of nucleic acid probes. In *Nucleic Acid Techniques in Bacterial Systematics* (Stackebrandt, E. and Goodfellow, M., eds.). John Wiley & Sons Ltd., Chichester, United Kingdom, 205–248.
